# Trends in Health Quality–Related Publications Over the Past Three Decades: Systematic Review

**DOI:** 10.2196/31055

**Published:** 2022-10-04

**Authors:** Joseph Mendlovic, Francis B Mimouni, Iris Arad, Eyal Heiman

**Affiliations:** 1 Shaare Zedek Medical Center Jerusalem Israel

**Keywords:** health quality, publication, medline, quality assessmnet, healthcare quality

## Abstract

**Background:**

Quality assessment in health care is a process of planned activities with the ultimate goal of achieving a continuous improvement of medical care through the evaluation of structure, process, and outcome measures. Physicians and health care specialists involved with quality issues are faced with an enormous and nearly always increasing amount of literature to read and integrate. Nevertheless, the novelty and quality of these articles (in terms of evidence-based medicine) has not been systematically assessed and described.

**Objective:**

The objective of this study was to test the hypothesis that the number of high-evidence journal articles (according to the pyramid of evidence), such as randomized control trials, systematic reviews, and ultimately, practice guidelines, increases over time, relative to lower-evidence journal articles, such as editorials, reviews, and letters to the editors.

**Methods:**

We used PubMed database to retrieve relevant articles published during the 31-year period between January 1, 1989, and December 31, 2021. The search was conducted in April 2022. We used the keywords “quality care,” “quality management,” “quality indicators,” and “quality improvement” and limited the search fields to title and abstract in order to limit our search results to articles nearly exclusively related to health care quality.

**Results:**

During this 31-year evaluation period, there was a significant cubic increase in the total number of publications, reviews, clinical trials (peaking in 2017, with a sharp decline until 2021), controlled trials (peaking in 2016, with a sharp drop until 2021), randomized controlled trials (peaking in 2017, with a sharp drop until 2021), systematic reviews (nearly nonexistent in the 1980s through 1990s to a peak of 222 in 2021), and meta-analyses (from nearly none in the 1980s through 1990s to a peak of approximately 40 per year in 2020). There was a linear increase in practice guidelines from none during 1989-1991 to approximately 25 per year during 2019-2021, including a cubic increase in editorials, peaking in 2021 at 125 per year, and in letters to the editor, peaking at 50-78 per year in the last 4 years (ie, 2018-2021).

**Conclusions:**

Over the past 31 years, the field of quality in health care has seen a significant yearly increase of published original studies with a relative stagnation since 2015. We suggest that contributors to this dynamic field of research should focus on producing more evidence-based publications and guidelines.

## Introduction

### Overview

Quality assessment in health care is a process of planned activities with the ultimate goal of achieving a continuous improvement of medical care through the evaluation of structure, process, and outcome measures [1-4].

Practicing physicians and health care administrators dealing with quality issues face a formidable challenge in following the developments of their fields of expertise, especially in view of the ever-increasing number of medical publications [5]. The number of medical journals have increased over the years and internet makes them readily accessible [5]. Several effective engines allow rapid searches for medical articles. The National library of Medicine offers PubMed as a free service. PubMed classifies publications by type, including clinical trials, editorials, letters, systematic reviews, meta-analyses, practice guidelines, randomized controlled trials, and reviews, among others. This classification is particularly important for researchers, as it provides the reader with some kind of ‘quality’ assessment in terms of evidence-based medicine. Evidence-based medicine classifies article as a pyramid that has articles of the lowest level of evidence (eg, expert opinions or background information) at its basis, and at its top, it has articles with the highest level of evidence (ie, systematic reviews) [6]. This pyramid is modified from time to time, as many suggestions are offered in order to improve it [7].

The number of publications in the field of health care quality predictably increases over time, in view of the constantly increasing number of journals, researchers, funding, the appearance of open access publications, and the technologic improvements in publication procedures and speed. It is important, however, to verify whether the quality of articles published in this field of medicine improves over time, as compared to their quantity. Physicians and health care specialists involved with quality issues are faced with an enormous and nearly always increasing amount of literature to read and integrate, but it is not clear whether the quality of these articles has also increased. To the best of our knowledge, novelty and quality of these articles (in terms of evidence-based medicine) has not been systematically assessed and described, and the objective of our systematic review of articles published in this field of medicine was to fill this gap. We, therefore, aimed to verify the hypothesis that there is a relative significant increase in the number of high-evidence journal articles, such as randomized control trials, systematic reviews, and ultimately, practice guidelines, in particular, as compared with ‘lower-quality’ (in terms of evidence-based classification) articles.

## Methods

### Digital Database

We used PubMed [8] to retrieve relevant articles published between January 1, 1989, and December 31, 2021. We followed the PRISMA (Preferred Reporting Items for Systematic Reviews and Meta-Analyses) requirements for a systematic literature review [9]. The search was conducted in April 2022. We focused on the field of health care quality in a manner similar to previous studies performed by us [10]. In order to do so, we attempted to retrieve as many health care quality–related articles as possible. We did not use additional databases, such as Embase or Google Scholar, because of the considerable amount of ‘noise’ added, that is, mostly articles published in nonprofessional journals. As a threshold of quality, we aimed to only look at articles published in journals registered in MEDLINE.

### Search Strategy

We searched for the following keywords: “quality care” OR “quality management” OR “quality indicators” OR “quality improvement.” A preliminary search conducted in this fashion returned a huge number of articles unrelated to health care quality, in which the word “quality” appeared several times in the body of the article, for example in the expression “quality of life.” Similarly, the addition of the search terms “quality control,” ”quality assessment,” and “quality assurance” retrieved even more articles, and the vast majority of these articles were not related to health care quality. Thus, in the final search, we used the keywords “quality care,” “quality management,” “quality indicators,” and “quality improvement” and limited the search field to title and abstract only, which allowed us to limit our search results to articles nearly exclusively related to health care quality.

### Inclusion and Exclusion Criteria

We aimed to include all articles related to quality in health care and exclude all those that were retrieved by the search but were not related to health care. In view of our systematic review of all titles and abstracts, 2 of the authors (JM and FBM) were able to verify that the keywords and search strategy allowed us to include all the articles retrieved without exclusion. We also limited the search to articles written in English and dealing with humans (as opposed to animals). We repeated the search by each time using one limit according to publication types as classified by PubMed, and we noted the total number of publications per year for the 31 years of the specified period. As mentioned in the introduction section, we used PubMed’s own classification of articles such as relatively ‘low-evidence articles’ (eg, case reports, editorials, letters, and reviews), and higher-evidence ones (eg, clinical trials, controlled trials, randomized controlled trials, meta-analyses, systematic reviews, and practice guidelines) [10,11]. In order to verify that the classification and tagging offered automatically by PubMed was accurate, we used a random sample of 5 articles each year, and in 100% of the cases, PubMed’s classification was accurate. There are, however, obvious overlaps; for instance, all randomized controlled trials are also classified as clinical trials; some papers, based on a case report and a review of the literature, are classified both as reviews and case reports; systematic reviews are also classified as reviews. Some but not all meta-analyses are part of systematic reviews.

### Statistical Analyses

The Minitab Statistical package (version 16.0; Penn State University) was used for statistical analyses. We used regression analysis (ie, linear and best-fit nonlinear) to determine the effect of year of publication on the number of publications of each type. A *P* value <.05 was considered significant.

## Results

**Figure 1:**
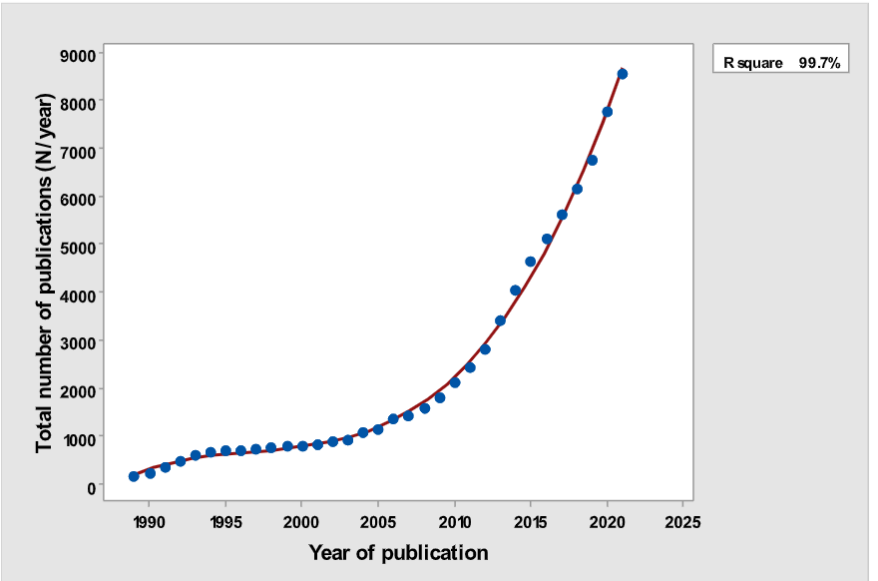
Total yearly number of publications (y-axis) versus year of publication (x-axis).

**Figure 2:**
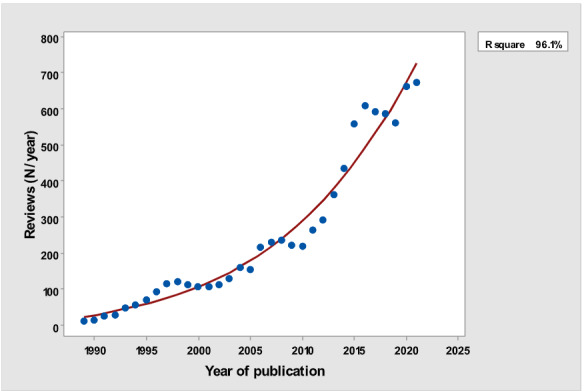
Yearly number of reviews (y-axis) versus year of publication (x-axis).

**Figure 3:**
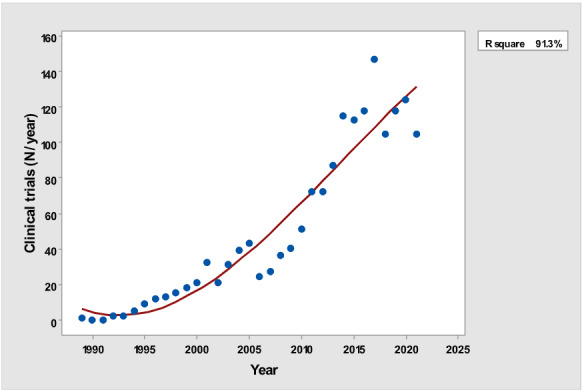
Yearly number of clinical trials (y-axis) versus year of publication (x-axis).

**Figure 4:**
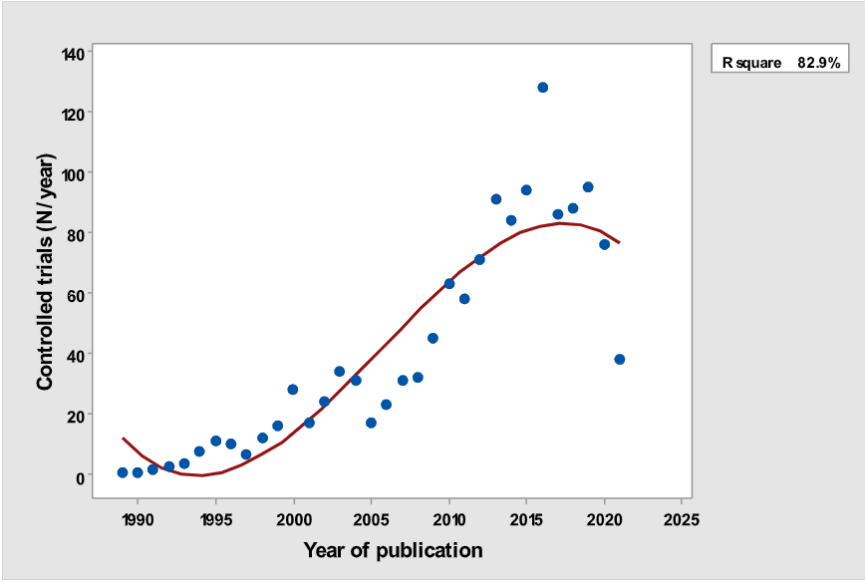
Yearly number of controlled trials (y-axis) versus year of publication (x-axis).

**Figure 5:**
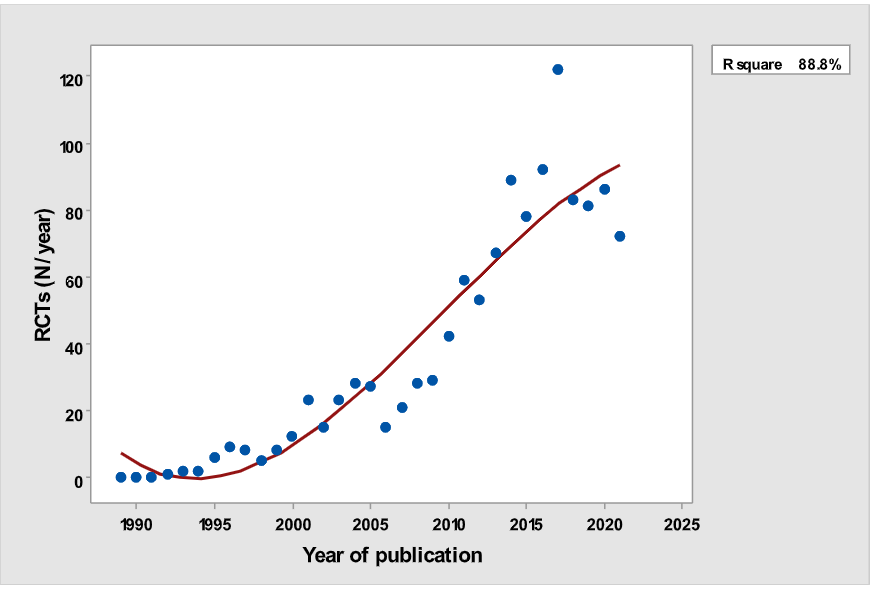
Yearly number of randomized controlled trials (y-axis) versus year of publication (x-axis). RCT: randomized controlled trial.

**Figure 6:**
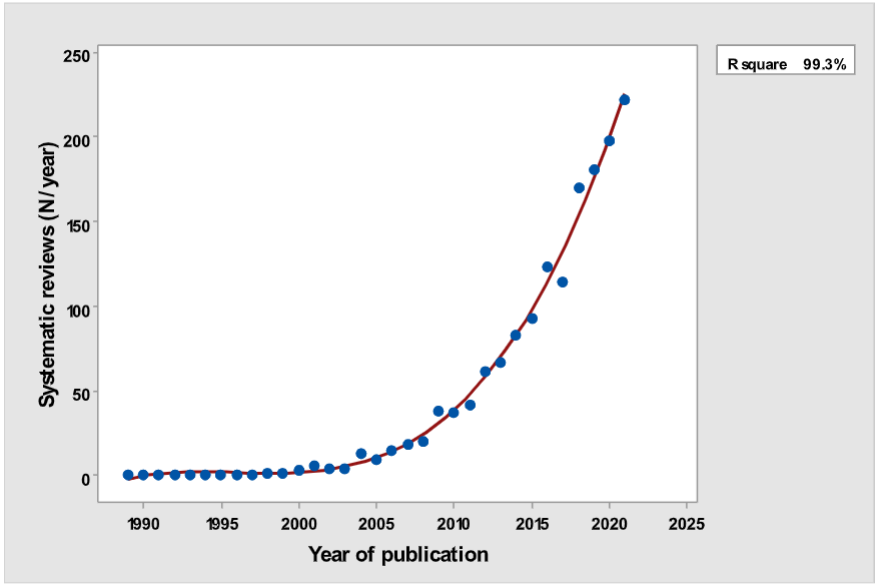
Number of Systematic reviews (y-axis) per year (x-axis).

**Figure 7:**
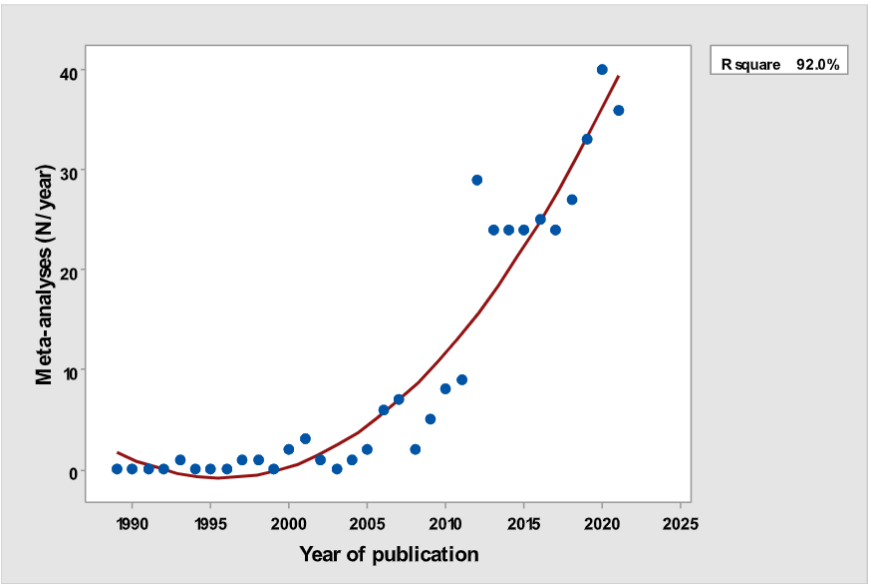
Number of Meta-analyses (y-axis) per year (x-axis).

**Figure 8:**
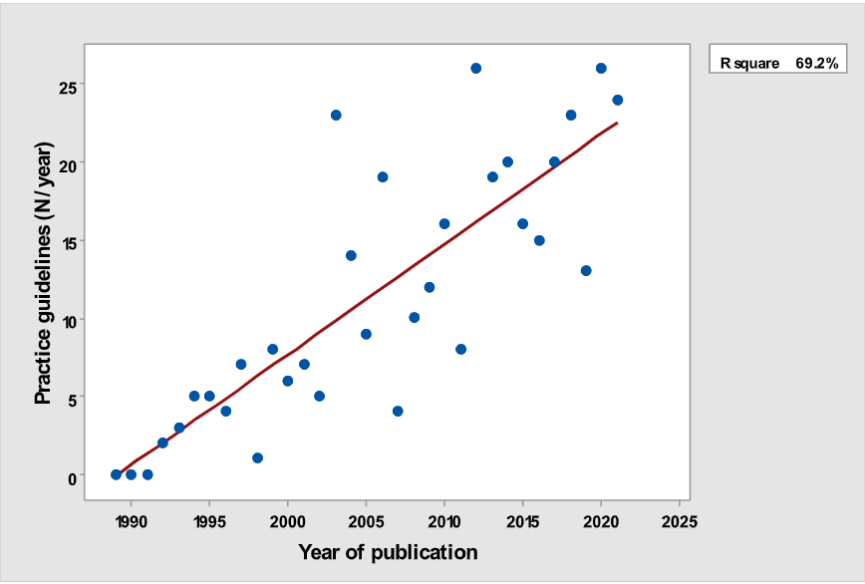
Number of practice guidelines (y-axis) per year (x-axis).

**Figure 9:**
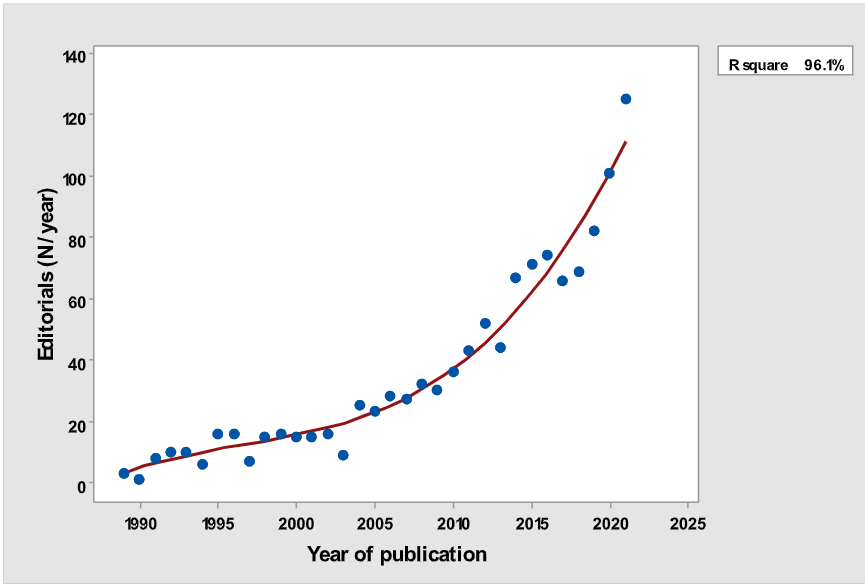
Number of editorials (y-axis) per year (x-axis).

**Figure 10:**
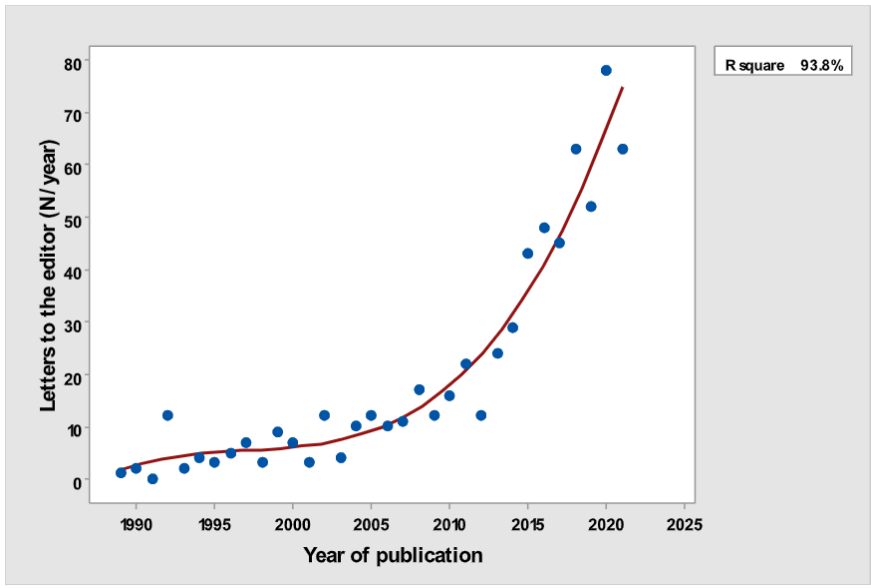
Yearly number of Letters to the editor (y-axis) versus Year of Publication (x-axis).

Table 1 depicts the number of each type of publication retrieved using the stratification of 7 different research and publication methods selected by year. There was a significant cubic increase over the study period in the total number of publications (*R*^2^=0.997; *P*<.001; Figure 1) ; reviews (*R*^2^= 0.961; *P*<.001; Figure 2); clinical trials (*R*^2^=0.822; *P*<.001), peaking in 2017, with a sharp decline until 2021 (Figure 3); controlled trials (*R*^2^=0.829; *P*<.001), peaking in 2016, with a sharp drop until 2021 (Figure 4); randomized controlled trials (*R*^2^=0.888; *P*<.001), peaking in 2017, with a sharp drop until 2021 (Figure 5); systematic reviews (*R*^2^=0.993; *P*<.001), from nearly none in the 1980s through 1990s to a peak of 222 in 2021 (Figure 6); and meta-analyses (*R*^2^=0.920; *P*<.001), from nearly none in the 1980s through 1990s to a peak of approximately 40 in 2020 (Figure 7); There was a linear increase in practice guidelines from none in 1989-1991 to approximately 25 per year during 2019-2021 (*R*^2^=0.692; *P*<.001; Figure 8); there was a cubic increase in editorials (*R*^2^=0.961; *P*<.001; Figure 9), peaking in 2021 at 125 per year, and in letters to the editor (*R*^2^=0.858; *P*<.001; Figure 10), peaking at 50-78 per year in the last 4 years prior to this study, from 2018 until 2021.

**Table 1:** Types of publication retrieved in this study.

Year	Case reports	Clinical trials	Controlled trials	Editorials	Letters	Meta-analyses	Practice guidelines	Randomized controlled trials	Reviews	Systematic Reviews	Total publications
2021	36	105	38	125	63	36	24	72	674	222	8543
2020	34	124	76	101	78	40	26	86	662	198	7773
2019	17	118	95	82	52	33	13	81	560	181	6738
2018	26	105	88	69	63	27	23	83	586	170	6158
2017	20	147	86	66	45	24	20	122	593	114	5622
2016	16	118	128	74	48	25	15	92	610	123	5095
2015	12	113	94	71	43	24	16	78	559	93	4647
2014	16	115	84	67	29	24	20	89	435	83	4033
2013	17	87	91	44	24	24	19	67	361	67	3396
2012	13	72	71	52	12	29	26	53	291	61	2798
2011	12	72	58	43	22	9	8	59	264	41	2407
2010	16	51	63	36	16	8	16	42	218	37	2110
2009	15	40	45	30	12	5	12	29	222	38	1793
2008	7	36	32	32	17	2	10	28	234	20	1577
2007	8	27	31	27	11	7	4	21	230	18	1408
2006	4	24	23	28	10	6	19	15	215	14	1357
2005	5	43	17	23	12	2	9	27	153	9	1120
2004	6	39	31	25	10	1	14	28	158	13	1064
2003	7	31	34	9	4	0	23	23	127	4	900
2002	4	21	24	16	12	1	5	15	111	4	865
2001	5	32	17	15	3	3	7	23	107	5	820
2000	6	21	28	15	7	2	6	12	107	3	774
1999	6	18	16	16	9	0	8	8	111	1	774
1998	4	15	12	15	3	1	1	5	120	1	747
1997	13	13	6	7	7	1	7	8	113	0	717
1996	8	12	10	16	5	0	4	9	91	0	677
1995	0	9	11	16	3	0	5	6	68	0	686
1994	8	5	7	6	4	0	5	2	55	0	655
1993	5	2	3	10	2	1	3	2	46	0	594
1992	2	2	2	10	12	0	2	1	28	0	447
1991	2	0	1	8	0	0	0	0	23	0	342
1990	1	0	0	1	2	0	0	0	14	0	197
1989	2	1	0	3	1	0	0		11	0	147

## Discussion

### Principal Findings

This study demonstrates that physicians and health care specialists involved with quality issues are faced with an enormous and nearly always increasing amount of literature to read and integrate. It is striking, however, that this amount may have shown a trend toward stabilization in important types of publications, such as clinical trials, controlled trials, and randomized controlled trials, or even a decrease since 2015.

The slope of the increase was not the same for each type of publication. The rate of yearly increase in the number of publications was the slowest for clinical guidelines (linear rather than cubic). The number of yearly clinical guidelines increased slowly over time, strikingly different from the quasi-exponential increase in the total yearly number of all publications. Letters to the editors and editorials continued to steadily increase over the years of the study. The greatest rate of increase was for reviews.

### Comparison With Prior Work

The trend toward stabilization in important types of publications, such as clinical trials, controlled trials, and randomized controlled trials, or even a decrease in these publications since 2015 might be unique to the field of health care quality, as the number of publications reported by the National Library of Medicine and registered in PubMed has increased exponentially over the same period without any such decline since 2015 [12].

The number of yearly clinical guidelines increased slowly over time, strikingly different from the quasi-exponential increase in the total yearly number of all publications. Practice guidelines are important in every field of medicine because they are to set up a standard based upon high level of evidence, and even if such type of evidence does not exist, they are at least based upon expert opinion. Graham et al [13] recently stated:

The most important benefit of clinical practice guidelines is their potential to improve both the quality or process of care and patient outcomes. Increasingly, clinicians and clinical managers must choose from numerous, sometimes differing, and occasionally contradictory, guidelines.

We can only speculate about this phenomenon. One possible explanation is that, as a rule, guidelines are to be followed. Not following them may lead to malpractice suits, and the fear for malpractice suits might be a deterrent for professional associations to publish such guidelines [14]. Guidelines also require an organizational infrastructure (eg, a professional association or academy), at a national or international level, that is capable of identifying an important and often controversial topic and will invest the necessary resources to enlist professional experts and often fund their time and travel expenses to a common meeting place, where the guidelines will be written. Such an infrastructure may not be established enough in the field of health care quality to allow for the development and subsequent publication of numerous guidelines every year [15]. Writing guidelines also requires reaching a consensus [15], and professional associations in the field of health care quality might not be organized enough to issue a large number of guidelines. Finally, there might be a limit of how many guidelines can be written in a particular field, and it is possible that the field of quality in health care might have reached some degree of ‘saturation’ in the number of potential guidelines.

Letters to the editors are usually author initiated, contrary to editorials, which are mostly invited. Nevertheless, these 2 types of articles continued to steadily increase over the years of the study, in spite of the fact that they are both unlikely to add much evidence to medical knowledge and rank very low in the evidence-based pyramid [7].

Reviews and systematic reviews are often written by invitation, and they have the potential for being highly quoted, in particular when there is a restriction in the number of references [16]. There was a fast increase in both types of articles, but we suspect that a relative stagnation in the number of randomized controlled trials will limit the ability to perform systematic reviews at increasing rates in the near future.

### Limitations

One limitation of our study is that we cannot claim that our search allowed us to recall all papers published in the field of health care quality. The inclusion of additional keywords or other languages may have added a substantial number of publications. In addition, quality is a broad concept with different dimensions, frameworks, and even definitions. Quality indicators are categorized into input, process, output, outcome, and impact. Each published study can focus on any one of these indicators. Moreover, quality studies are affected in various settings, including primary care, secondary, or tertiary settings. Some terms, such as “patient satisfaction” or “health marketing,” may in fact be related to some aspects of quality. Thus, the search strategy and classification that we used in this study may not show a true picture of the volume of studies in this field. However, we do not believe that accessing those articles would have significantly modified our findings or our conclusions, in view of the very large number of publications that we were able to retrieve. Another limitation of our study is that we did not use other databases such as Embase or Google Scholar. Adding these databases would have probably helped us retrieve additional articles, but they would likely be articles published in journals not registered in PubMed [17]. Some of them may well have been published in legitimate, ‘newer’ journals not yet registered in MEDLINE, but as a threshold of quality, we aimed to only look at those registered in MEDLINE, which our methodology allowed us to do.

Another limitation of our study is that the classification and tagging offered by PubMed may not be 100% accurate. This applies mostly to the type of study. Classification errors are probable. However, a random sample of the retrieved articles revealed an excellent degree of agreement with PubMed classifications. Moreover, although the number of health quality–related papers appeared to be rising in quantity, we could not determine whether it also increased in quality, since PubMed does not classify medical articles by quality. At times, a few articles of very high quality will have a much more meaningful impact on clinical care than many other articles of lesser quality. Our study somewhat warns physicians and health care specialists who wish to address health care quality issues that many reviews and commentaries in this field may be poorly supported by solid evidence.

### Conclusions

Over the past 29 years, the field of quality in health care has seen a significant annual increase of published original studies, with a relative stagnation or decrease since 2015. As the internet has created a revolution in the availability and accessibility of scientific publications, it may yet create additional striking changes in the trends that we currently report. Moreover, secular changes in funding priorities may also create significant changes in the future. We suggest that contributors to this dynamic field of research should strive to produce more evidence-based publications and guidelines rather than commentaries and nonsystematic reviews that do not really provide much additional evidence.

As digital health includes concepts from an intersection between technology and health care, we hope that in the years to come, there will be digital transformations to the health care field that will enable researchers, practicing physicians, and health administrators to have better and faster means to find evidence-based solutions for the quality problems they face.

## Abbreviations

PRISMA: Preferred Reporting Items for Systematic Reviews and Meta-Analyses
